# Recent Advances in Biopolymeric Membranes towards the Removal of Emerging Organic Pollutants from Water

**DOI:** 10.3390/membranes11110798

**Published:** 2021-10-20

**Authors:** Feziwe B. Mamba, Bhekani S. Mbuli, James Ramontja

**Affiliations:** 1 Department of Chemical Sciences, Faculty of Science, University of Johannesburg, P.O. Box 17011, Doornfontein, Johannesburg 2028, South Africa; feziwemamba@gmail.com; 2 DST/Mintek Nanotechnology Innovation Centre, University of Johannesburg, Johannesburg 2028, South Africa; 3 Centre for Nanomaterials Science Research, University of Johannesburg, Johannesburg 2028, South Africa

**Keywords:** membrane fouling, micropollutants, water treatment, separation mechanisms, biodegradable

## Abstract

Herein, this paper details a comprehensive review on the biopolymeric membrane applications in micropollutants’ removal from wastewater. As such, the implications of utilising non-biodegradable membrane materials are outlined. In comparison, considerations on the concept of utilising nanostructured biodegradable polymeric membranes are also outlined. Such biodegradable polymers under considerations include biopolymers-derived cellulose and carrageenan. The advantages of these biopolymer materials include renewability, biocompatibility, biodegradability, and cost-effectiveness when compared to non-biodegradable polymers. The modifications of the biopolymeric membranes were also deliberated in detail. This included the utilisation of cellulose as matrix support for nanomaterials. Furthermore, attention towards the recent advances on using nanofillers towards the stabilisation and enhancement of biopolymeric membrane performances towards organic contaminants removal. It was noted that most of the biopolymeric membrane applications focused on organic dyes (methyl blue, Congo red, azo dyes), crude oil, hexane, and pharmaceutical chemicals such as tetracycline. However, more studies should be dedicated towards emerging pollutants such as micropollutants. The biopolymeric membrane performances such as rejection capabilities, fouling resistance, and water permeability properties were also outlined.

## 1. Introduction

The conventional wastewater treatment plants (WWTPs) have demonstrated to have limited effectiveness against the removal of these CECs. In such cases, the treated WWTP effluents tend to contain the organic micropollutant contaminants [1]. For example, Nam et al. [1] reported that nonylphenol, an endocrine disruptor, had limited removal efficiency, ranging between 53% and 55%, within WWTPs; consequently, the nonylphenol is detected in treated WWTP effluents [1]. Emerging contaminants have been overlooked for the longest time and limited studies have been conducted on their detection and remediation as emerging contaminant threats [2,3]. This is especially because few researchers are concerned about the adverse effects of micropollutants. Therefore, novel technologies dedicated to the remediation of the organic micropollutants should be developed [2]. 

Hence, in this review, membrane technology is discussed to cater for its challenges and success in the removal capacity of organic micropollutants from water. Notably, secondary pollution occurs because of the disposal of the remnants of membranes and/or adsorbents after use. Unfortunately, these plastics that remain further pollute the available water suitable for drinking. This occurs because the materials being used to treat water are disposed into landfills and may further pollute groundwater. As such, most polymeric membranes are not degradable and turn out to have extended half-life and this may be detrimental to the environment. However, secondary pollution can be eliminated by using biodegradable material for micropollutant remediation. Herein, this review paper discusses the utilization for biopolymers in membrane technology applications. Therefore, this paper comprehensively reviews the recently reported membrane filtration-based techniques specifically dedicated towards organic micropollutants’ water treatment. Fundamentally, implementation drawbacks of biopolymeric membranes in water purification are discussed. Further to this, strategies that can improve biopolymeric membrane properties are discussed.

### 1.1. Biopolymers: Properties and Applications

Biopolymers are polymers that are relatively sourced from living organisms, such as plants and microbes, rather than from petroleum [4]. They are classified as synthetic and natural biopolymers [5]. The synthetic biopolymers are polymers that can either be modified from natural polymers or chemically synthesized from synthetic monomers. For example, synthetic biopolymers include polylactic acid (PLA), polycaprolactone (PCL), and polyvinyl alcohol (PVA). On the other hand, natural biopolymers are sourced from natural sources [5,6]. These naturally sourced biopolymers include cellulose, starch, chitosan [5,6,7], collagen, fibrinogen [5,6], chitin, alginate, and carrageenan, among others [7]. Among these, chitosan and cellulose are the most readily available biopolymers in nature. Carrageenan, which is a seaweed, is divided into three derivatives, i.e., kappa-carrageenan, iota-carrageenan, and lambda-carrageenan, which is determined by the degree of sulfation. Starch is mostly obtained from stalks, roots, and crop seeds [7]. Similarly, cellulose is sourced from plants, fungi, bacteria, and algae [8]. Cellulose has been extracted from various agricultural waste materials such as pomelo [9], banana peels, corn stalks, vegetable waste, wood chips, grain husks, stubble [10], peels from mango, cucumber [11], oranges [11,12], banana [10,11], bagasse, nut shells, willow branches, rice husks, and straw [12].

Biopolymers are hydrophilic, and this enhances membrane-fouling resistance and water permeability when utilised as membranes for water treatment [8,13]. The hydrophilic functional groups preferentially enable the formation of a thin layer of water on membranes’ surfaces. This preferential formation on the hydrophilic membrane surfaces prevents the deposition of foulants. Thus, this phenomenon reduces membrane fouling [13]. Furthermore, cellulose is highly hydrophilic, with contact angles ranging between 20° and 30°. Fundamentally, the hydrophilic properties of the cellulose are brought about by the presence of hydroxyl functional groups. These hydroxyl functional groups form hydrogen bonds with water, and this enables preferential attachment of water molecules that promote water permeability [8]. Furthermore, biopolymers are highly considered because of their ability to decompose once disposed in landfills [5]. Fortunately, there are no harmful by-products that are released during the biodegradation of biopolymers. The known by-products include humus, carbon dioxide, biomass, and methane. Thus, it becomes advisable to utilize biopolymers in water treatment applications [10].

### 1.2. The Use of Biopolymers in Membrane Techniques

Various researchers have developed several approaches towards the remediation of wastewater treatment. These techniques include adsorption, filtration, membrane technology, and advance oxidation methods such as ozonation, photocatalysis [14,15], and biodegradation [15]. However, these technologies have to be highly effective, efficient, and economical [16]. Membranes are preferred over conventional water treatment processes, such as flocculation, adsorption, and coagulation, among others, because membranes have demonstrated effective wastewater purification and they pose limited hazard to the environment [17]. During separation, a membrane acts as a barrier to selectively allow water molecules to pass through and prevent the passage of impurities [18]. A wide variety of polymers have been used in the fabrication of non-biodegradable polymeric membranes. Notably, most commercial membranes are synthesized and fabricated using non-biodegradable materials. The commonly used non-biodegradable polymers include polyvinylidene fluoride (PVDF) [13,19,20,21], polysulfone (PSf), polyethersulfone (PES) [19,20,21,22], polyphenylsulfone [22], polypropylene [23], polyvinyl alcohol, polystyrene, and poly(1,5-diaminonaphthalene), among others [24]. Most of these synthetic polymers used in membrane production are non-biodegradable and possess hydrophobic properties. Regrettably, the hydrophobicity of these membranes renders them susceptible to membrane fouling [13,19,22]. Unfortunately, once the micropollutant foulants are deposited on the membrane surfaces, the water permeability is compromised. Consequently, this increases the need for frequent membrane cleaning, thereby increasing maintenance costs of the membrane system [13,19,22,25].

Notably, fouling increases operational costs because of constant membrane cleaning, usually accomplished through chemical washing. Consequently, the quality of the membrane and lifespan get reduced significantly [18,25]. In contrast, biopolymers are renewable, sustainable, biodegradable, cost effective, compostable, eco-friendly, non-toxic, biocompatible, and hydrophilic [26,27,28,29,30,31]. Furthermore, biopolymers are known to be hydrophilic, and hydrophilic materials significantly improve the rejection capacity and efficiency of biopolymeric membranes [26,27,32,33,34]. Thus, biopolymers can substitute the potentially toxic and nonbiodegradable polymers used as an alternative in membrane fabrication since they are environmentally friendly [26,27,32].

It is highly desirable that methods to remove and neutralize organic micropollutants be efficiently developed [35]. For example, membrane filtration technologies have been considered because of their inherent simplicity and efficiency, even at high pollutant concentrations. Furthermore, they have minimal solid waste generation. In addition, membranes tend to eliminate virtually all types of dyes, salts, and mineral derivatives [36]. Therefore, membrane technology has promising capacity towards organic pollution treatment [37]. There are different membrane technologies, and these are ultrafiltration (UF), nanofiltration (NF), reverse osmosis (RO), and forward osmosis (FO) membranes. These membrane technologies can remove numerous micropollutant contaminants. For instance, nanofiltration and reverse osmosis membranes are commonly utilized to treat micropollutants and they have demonstrated some level of efficiency towards micropollutant remediation [35,38]. For example, NF membranes have been used for the retention of multivalent ions and organic compounds under relatively low operation pressure [37]. On the other hand, RO membranes can remove monovalent salts and micropollutants from water, even at low concentrations.

Polymeric membranes are the commonly utilized materials during membrane fabrication because of their ease to work with. Consequently, membrane fabrication processing parameters are formulated and implemented to accomplish maximum water permeability and micropollutant rejection performances [39]. As such, the removal efficiencies of these polymeric membranes can be experimentally controlled through adjusted contact time parameters, type of water sources, concentrations of micropollutants, and building components of the membranes [2].

These non-renewable and non-biodegradable polymers can be detrimental to the environment over time. This is because after their service half-life, they are usually discarded onto landfills and sometimes find their way into the aquatic environment. In some case, these materials are burned, thereby leading to secondary pollution such as global warming [19,40]. As such, environmental pollution and landfills are currently stretched beyond capacity. Thus, adding non-biodegradable membrane materials on top of the environmental pollution pressure becomes detrimental. Therefore, there is a need to develop completely biodegradable materials that can easily degrade completely after use, thereby preventing secondary pollution. Natural biopolymers such as starch, pullulan, cellulose, chitosan, alginate, and proteins have been used in water purification [6]. Such polymers have been sourced from waste material and converted into reusable materials such as cellulose, thus reducing waste material [10].

### 1.3. Water Micropollutants and Their Impact on Human and Animal Health

One challenge in water treatment of micropollutant-contaminated water is the change of chemical structures into new chemical moieties, which might be even more toxic than the parent organic compound [14]. Furthermore, the organic micropollutant contaminants in water tend to resist degradation; hence, such pollutants turn out to be persistent in water. Consequently, these chemicals bioaccumulate in adipose tissues of aquatic animals. This is, therefore, detrimental to the aquatic life’s health [15].

Organic micropollutants are organic compounds that are toxic to the environment within water bodies, even at low concentrations, ranging between μg/L and ng/L levels [41,42]. Prevalent micropollutants’ contamination such as polyaromatic hydrocarbons, antibiotics, pesticides, contraceptive medicines, and personal care products have been deposited into freshwater sources, thus leading to their accumulation in water bodies [41]. Some of the organic pollutants are released from agricultural activities, wastewater discharge from industries or households, accidental chemical spills, and oil spillage [41,43]. Notably, these include pesticides, pharmaceuticals, cosmetics, flame retardants, perfumes, waterproofing agents, plasticizers, and insulating foams, among others [42,44,45]. The commonly detected pharmaceuticals are antibiotics, antidepressant, antiepileptic, anti-inflammatory, and antiretroviral drugs [2,44]. Furthermore, these organic pollutants are known to be hazardous and noxious substances to the environment such as aquatic life and terrestrial animals dependent on those water body systems [41,43]. Due to such water pollution, the scarcity of clean and safe drinking water from natural sources remains on the rise [41,46].

**Figure 1:**
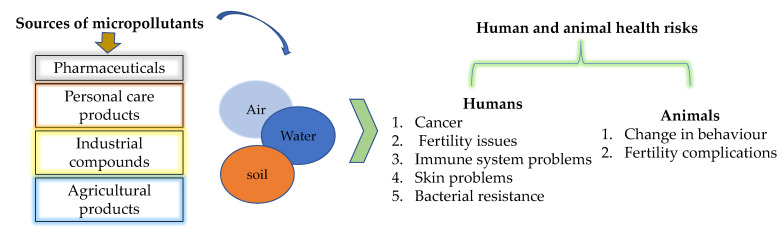
Some of the sources of micropollutants and their effects on humans and animals, as adapted from Vasilachi et al. [51].

Unfortunately, even at low concentrations, organic micropollutants have been found to be detrimental to aquatic animals and human beings, as illustrated in Figure 1 [41]. For example, human beings’ exposure to pesticides such as organophosphates leads to challenges such as cognitive defects, cancer, and mutagenic complications [45,47]. Furthermore, elongated exposure to atrazine can inhibit androgenic hormone-related development, thus negatively affecting fertility in men and increasing chances of breast cancer in women [41].

Other conditions related to elongated exposure to organic micropollutants include skin blisters, respiratory challenges, and eye burns, and extreme cases may result in fatigue, lung problems, immune system damage, and cancer [48]. Furthermore, unregulated consumption of hormones can lead to the decrease of male sperm count. This may also upsurge cases of testicular, prostate, ovarian, and breast cancer, and effectively increase occurrences of reproductive malfunctions in human beings. Notably, endocrine disruption, genetic make-up damage, resistance in pathogenic bacteria, and aquatic toxicity are also associated with organic micro-pollution challenges [46,49].

Additionally, the development of bacterial resistance makes common infections, minor injuries, and routine operations riskier due to unmonitored consumption of antibiotics in drinking water, another concern to health systems [45]. Furthermore, long exposure of organisms to the organic micropollutants can affect the health of aquatic life such as the change in behavior of fish, which tends to affect their aggression, reproduction, and feeding activities [50].

As industries are being developed and their products’ demand remains high, there is an increased usage of organic compounds, which leads to the high demand of water purification and treatment technologies. As it is, the increased utilization of organic products such as pharmaceuticals and pesticides has resulted in the reduction of clean water supplies for communities [52]. Furthermore, these contaminants of emerging concerns (CECs) tend to find their way into water systems [1,53,54]. As such, the most abundant agricultural contaminants include pesticides and herbicides such as atrazine, endosulfan cyanazine, and metribuzin. Notably, industries usually release these chemicals, such as methyl-tert-butyl-ether, and aromatics, such as benzene, toluene, dichlorobenzenes, and xylenes. Furthermore, personal care products that find their way into the water systems include caffeine, trimethoprim, sulfamethoxazole, carbamazepine, and diclofenac [53].

**Figure 2:**
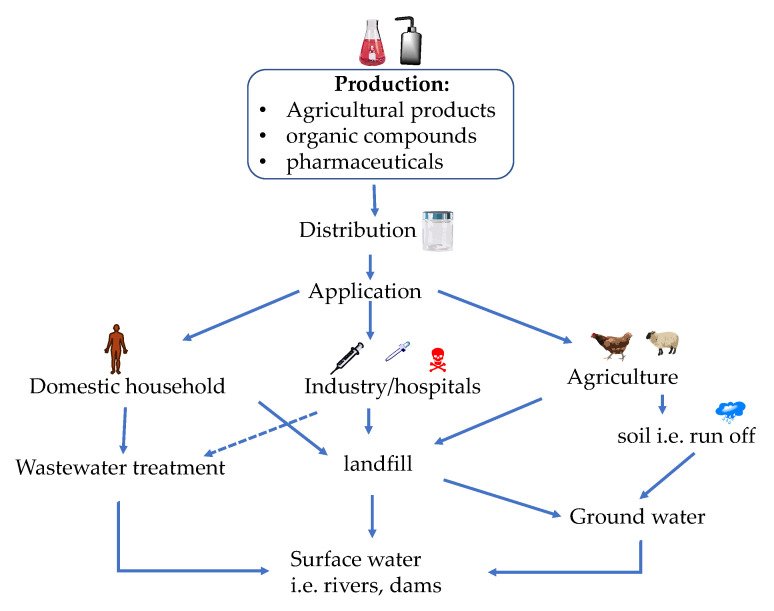
A path of micropollutants from production to drinking water, as adapted from Kanaujiya et. al. and Kümmerer et.al., respectively [55,56].

These organic micropollutant contaminants find their way into water systems via various routes such as sewage treatment plants, human excretion [50], disposal of unused and expired pharmaceuticals, and agricultural pesticides [1,50]. In addition, the pollutants can also pass through drainage systems [1]. These micropollutants accumulate over time and are being progressively detected in water systems [1,54]. The sources of micropollutants and the various ways in which micropollutants find their ways into the water system are summarized in Figure 2.

### 1.4. Policy Frameworks and Guidelines for Water Treatment in South Africa

Commercially, the predominantly utilized polymeric membranes are non-biodegradable for storage and water treatment. Regrettably, these plastic membranes are characterized by long half-lives and barely degrade completely over time. Instead, these non-biodegradable polymeric membranes are broken down into microplastics (0.1 μm–5 mm) via various chemical and mechanical paths [57,58]. Unfortunately, these microplastics become an environmental concern as they persist on land and water systems, thereby causing secondary pollution.

South Africa and the rest of the world are currently struggling with these plastics polluting the environment. Notably, South Africa is among the leading contributors towards plastic pollution [59]. Therefore, using non-biodegradable membranes exacerbates this pollution problem. Hence, there is a need to eradicate this problem by seeking degradable membrane material that can easily be disposed and degrade after use. However, these biodegradable materials should be strong enough to complete the water treatment life cycle and effectively treat water. In most cases, researchers avoid using biodegradable materials because of mechanical strength concerns.

These microplastics have negative impacts on the environment and are harmful towards aquatic life. For example, microplastics have been reported to negatively impact marine life such as mussels in Cape Town, South Africa, as reported by Sparks et al. [57]. Therefore, relevant policies to address this plastic pollution challenge should be formulated, updated, implemented, and enforced to accommodate emerging micropollutants.

Remarkably, there are policy frameworks that have been established to control the level of pollution around the world, more especially in South Africa. For instance, the South African Water Guidelines and World Health Organization (WHO) have placed the atrazine detection limit concentration in water at 0.1 µg/L [60]. In other words, this implies that once the atrazine is above the limit, the contaminated water is regarded as unsuitable for human use as this may cause detrimental health challenges. Therefore, this calls for better water treatment technologies to address these contamination levels. However, such treatment technologies should be sustainable and avoid causing secondary pollution in the environment. This can be accomplished by adopting biopolymeric materials.

It is the United Nations’ and African Union’s goal to ensure that clean water and sanitation is provided for all [61,62]. Therefore, to meet the United Nation’s Sustainable Development Goal Number (SDG) 6 and corresponding Africa Union’s (AU) Agenda 2063 aspirations of providing clean water and sanitation for all, the South African Government established water contamination regulations that, when adhered to, can effectively prevent water pollution [62]. Such guidelines enable South African industrial activities to be regulated, to obtain an effluent discharge permit before releasing water into water systems. Failure to comply to such conditions of the permit can lead to penalties that include financial fines and subsequent withdrawal of operation permits [62,63].

Unfortunately, with all these policies and the challenges that are addressing the micropollutant levels, there are limited regulations that control the material used to treat water pollution. This includes addressing microplastics and possible secondary pollution brought about by using plastics/polymeric membranes to treat wastewater. As such, these regulations are not fully standardized, synchronized, and structured. Furthermore, the utilization of biopolymeric and degradable materials remains insufficiently encouraged by the South African government.

The South African government, through the Department of Environmental Affairs, is allowing plastic manufacturers to continue producing plastics with a minimum thickness of 24 microns, more especially for storage applications and purposes. Consequently, these plastics are highly produced for public use, even though it is at a price. Since the plastic price is significantly small (i.e., between R0.25–R0.80 per plastic bag), the public is still using these plastics ungoverned; hence, the uncontrollable plastic waste [64,65]. This clearly demonstrates that the plastics pollution challenge will not be addressed if researchers and innovators are not compelled to urgently consider biodegradable polymeric plastics as an option to explore for applications such as storage and water treatment, among others. Therefore, to fully realize environmentally friendly and sustainable water treatment, considerations should be made towards adopting biodegradable materials for water treatment applications. As such, the accompanying and enabling policies towards using biodegradable polymeric materials should be considered, formulated, and implemented to protect the environment. Consequently, creating these biopolymeric-biased policies can help South Africa realize the AU’s Agenda 2063 and UN’s SDGs.

## 2. Polymeric Membranes in Water Treatment

Membranes can either be polymeric or ceramic [21,52,66]. Notably, ceramic membranes are made from materials such as aluminum oxide (Al_2_O_3_) [52]. Ceramic membranes are known to possess excellent mechanical and thermal properties and possess significant chemical stability. In addition, ceramic membranes possess superior fouling resistance that can lead to extended membrane longevity [67]. Thus, such membranes can withstand the strong oxidizing agents and can therefore be utilized in large organic materials such as dyes’ removal applications [68]. Nevertheless, ceramic membranes have lower retention capacity on the removal of smaller organic materials [54,67]. This is because ceramic membranes have larger pores. Hence, ceramic membranes tend to limitedly retain micropollutants [54]. To this end, polymeric membranes are adopted to remove even small organic pollutants, including emerging micropollutants.

Polymeric membranes are advantageous over ceramic membranes because of their affordability and pollutant removal effectiveness [21,52]. Notably, polymeric membranes are known to be flexible. Thus, polymeric membranes can easily be fabricated for varied water treatment applications and environments. In addition, the polymeric membrane pore sizes are tunable when compared to ceramic membranes. Hence, the polymeric membranes can be applied in various industrial treatment applications towards the removal of smaller ions and trace elements from water [21,52]. Different polymeric membrane types can be used for water pollution filtration. This includes nanofiltration membranes that are applied in the filtration of brackish water, desalination, wastewater, and food industrial water treatments. Nanofiltration membranes are primarily used for the separation of divalent salts, heavy metals, and organic micropollutant molecules [33]. Reverse osmosis membranes, on the other hand, can remove monovalent salts from water. Ultrafiltration membranes can remove large organic molecules from water [69].

Polymeric membranes are mainly poor in temperature and chemical resistance compared to ceramic membranes and this interferes with the operational lifespan [52,70]. Thus, polymeric membranes with high chemical and thermal stability need to be developed to increase their usability under extreme conditions [70]. Pulido et al. [70] exhibited that pristine poly(oxindolebiphenylylene) membranes are chemically instable. Hence, crosslinkers were introduced to improve the stability of these membranes. The crosslinkers used for modification were 1-bromo-octane, 8-dibromo-octane, 1,4-di-bromo-butane, 1,4-diiodo-butane, 1,4-dibromo-2,3-butane-dione, α,α′-dibromo-p-xylene, and 1,5-dibromo-1,1,2,2,5,5-hexa-fluro-pentane. After crosslinking poly(oxindolebiphenylylene) with the different crosslinkers, the membranes were found to be stable against chemicals such as dimethyl sulfoxide (DMSO), N-methyl-2-pyrrolidone (NMP), dimethylformamide (DMF), and dimethylacetamide (DMA). Furthermore, Ba et al. [71] achieved chemically stable P84 copolyimide membranes during salt separation, either under acidic or basic conditions (i.e., 2 ≤ pH ≤ 10). These P84 copolyimide membranes were also stable against various organic solvents because of the imine functionality from the poly-ethylenimine.

**Figure 3:**
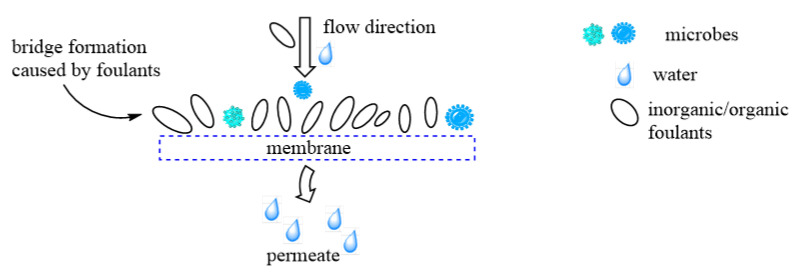
Membrane fouling mechanism during filtration [21].

The most common limitation of polymeric membranes is membrane fouling [23,72], as illustrated in Figure 3. During membrane fouling, a bridge (cake layer) is formed by organic and inorganic particles that turn to close the membrane pores. Subsequently, as the water sample flows through or across the membrane matrix, the foulants turn to be deposited onto the membrane surface and are sometimes trapped within the membrane pores. Consequently, these membrane pores turn to collect foulants until saturated, thereby forming a cake layer [21,73]. In addition, membrane fouling can also be caused by microorganism growth on membrane surfaces, also referred to as biofouling. Biofouling tends to grow on the surface of the membrane and subsequently formulates biofilm fouling [21,72,73].

Membrane fouling reduces water permeability in membranes [74]. Subsequently, the fouled membranes require high pressure to enable water to pass through the membrane’s matrix. Consequently, higher energy consumption is needed to enable the filtration process. On another hand, fouling delays filtration processes on operational membranes because of the sophisticated, frequent, and lengthy cleaning protocols. Fundamentally, this causes the use of the membranes to become more expensive. Most importantly, the membranes’ structural and tensile strength integrity turn out to be negatively impacted. Subsequently, this operational expenditure becomes costly because of the frequent replacement measures [23,74].

The hydrophilicity of membrane materials contributes significantly towards membrane antifouling properties. Notably, higher membrane hydrophilicity may prevent foulants’ deposits on membrane surfaces [21,73]. Other factors that affect the fouling of the membrane include the size of pollutants against membrane pores, shapes of pollutant particles in water samples, membrane’s porosity, and membrane surface’s functionality and charge [73]. As such, Katsoufidou et al. [74] demonstrated the fouling phenomenon by using sodium alginate. In this case, the researchers attributed the fouling of membranes because of calcium ions’ adsorptive binding to the polysaccharide functional groups. This resulted in cake development on the membrane surface [74]. Unfortunately, this cake layer ought to be cleaned constantly to regain the filtration efficiency of the membrane.

Lee et al. [72] demonstrated salt cleaning and osmotic back-wash studies on calcium-bridged, organic-fouled commercial flat sheet polyamide/polysulfone NF membranes. This was aimed at demonstrating cleaning possibilities of both techniques. The research study demonstrated that salt cleaning and osmotic backwash effectively cleaned the calcium-bridged, organic-fouled membranes. However, the osmotic backwash cleaning method had higher cleaning efficiency [72]. Even though the membranes can be cleaned, it still becomes an extra requirement that turns out to be costly and compromises the lifespan of the membranes. Hence, developing sustainable self-cleaning and antifouling membranes should be developed.

### 2.1. Removal of Emerging Organic Pollutants with Non-Biodegradable Polymers

Since these synthetic polymers are predominantly hydrophobic, there is always a need to modify with hydrophilic materials. In some cases, these synthetic polymers are blended with biopolymers, such as hydrogels, chitin, cyclodextrins, and nanoparticles, to introduce hydrophilicity, thereby improving water permeability and fouling resistance properties. For instance, in an attempt to increase the membrane hydrophilicity, graphene oxide (GO) nanocomposites have been explored. For example, Leaper et al. [75] fabricated PVDF membranes incorporated with superhydrophobic polyhedral oligomeric silsesquioxane with graphene oxide (POSS-rGO). Consequently, this hybrid POSS-rGO/PVDF membrane rendered stable water permeability and rejection as compared to the pristine PVDF membranes. Similarly, Nawaz et al. [76] incorporated polyaniline-GO into PVDF membranes. This resulted in improved hydrophilicity, which subsequently enhanced the pure water flux and antifouling properties and increased dye rejection of 95 % for methyl orange and 98 % for allura red compared to pure PVDF with a dye rejection of less than 30% of both dyes. Similarly, Vatanpour et al. [77] incorporated nitrogen-doped GO into PES membranes to enhance the membrane’s porosity, hydrophilicity, and their ability to experience hydrogen bonding during water filtration, hence, an improved rejection capacity of 91.1–95.6% as compared to the bare PES, which rejected 88.6% of Reactive Red 195 dye.

On the other hand, nanoparticles such as zirconium, metal organic frameworks, and faujasite have been used to improve the membrane hydrophilicity. For instance, Abdulkarem et al. [78] incorporated zirconium phosphate nanoparticles to improve the membrane hydrophilicity and water permeation. Dehghankar et al. [79] incorporated zirconium 1,4-dicarboxybenzene (UiO-66) and chromium (III) terephthalate (MIL-101) metal-organic frameworks (MOFs) and faujasite (FAU) zeolite nanocrystals into PVDF membranes. Consequently, this influenced the hydrophilic nature of the modified membranes, as demonstrated by the low water contact angles reported. Additionally, the porosity of the composite membrane was improved. The produced composite membranes had porosity% of 65–80%, whereas the neat membrane’s porosity was 65%. Palygorskite-chitin (PAL-CH) nanomaterials were incorporated by Mamah et al. [80] onto polyamide thin film composite membranes. Mutharasi et al. [81] incorporated Co-Al layered double hydroxide (LDH) into polysulfone membranes via coating.

In an attempt to enhance the selectivity of the PVDF, Altintas et al. [49] functionalized molecularly imprinted polymers with nanoparticles. As a result, the modified membranes exhibited 99.6% uptake capacity of 60.39 ng/cm^2^ for metoprolol, 94.7% uptake capacity of 45.09 ng/cm^2^ for diclofenac, and 42.6% uptake capacity of 16.9 ng/cm^2^ for vancomycin. On the other hand, Balta et al. [82] demonstrated that the addition of zinc oxide (ZnO) nanoparticles into PES polymeric membranes enhanced the membrane’s hydrophilicity. Furthermore, the PES/ZnO membranes demonstrated better water permeabilities and dye rejection when compared with neat PES membrane. This was attributed to the increased hydrophilicity of the ZnO-modified membranes.

Li et al. [83] illustrated that the addition of titanium dioxide (TiO_2_) nanoparticles onto PES membranes increased their hydrophilicity. As such, the researchers reported that the addition of the TiO_2_ nanoparticles augmented the hydrophilic properties of the membranes, as the formation of the pores. Such nanoparticle-induced properties enhanced the water permeability performances of the modified composite membranes. The effect of TiO_2_ nanoparticle sizes on the performance of PVDF membranes was also investigated by Cao et al. [84]. These researchers demonstrated that TiO_2_ nanoparticles with smaller radii introduced better the antifouling property onto the PVDF membranes. Consequently, the small-sized TiO_2_ nanoparticles onto PVDF membranes had smaller mean pore sizes and limited roughness properties on the membrane surfaces.

All these modified synthetic membranes have demonstrated superior water permeation and fouling resistance properties. Notably, the modifying and blending of synthetic polymers for water treatment applications significantly improves performance properties. However, their disposal after use and potential secondary pollution remains a challenge as such is compounding the already difficult plastics’ challenge the rest of the world is dealing with. Thus, considerations on exclusively utilizing biopolymers for water treatment applications should be made.

### 2.2. Environmental Impact of Synthetic Polymers

Non-biodegradable polymers have been used for water filtration, owing to their extreme chemical and thermal stability. However, their implementation can be detrimental to the environment [23]. For instance, during the synthesis of the polymers, the reagents are not completely reacted, such as styrene, caprolactone, bisphenol-A, acrylics, methacrylics, styrene, and vinyl acetate catalysts. This is due to interfering side reactions such as transesterification and ester/ester exchange. Hence, the conversion of the monomers into polymeric structures is often not 100% [85]. Consequently, these unreacted monomers may further pollute the environment [23]. Non-biodegradable polymers tend to break down into their building blocks’ monomers that might be toxic to the environment. For instance, polycarbonate polymers can degrade into bisphenol-A (BPA) when exposed to salty aquatic environments [86]. Unfortunately, the BPA is associated with cancer in humans as a threat to human health [19]. Furthermore, once polymeric membranes have been utilized, they are discarded and disposed onto landfills, and may also pollute water body systems. However, since the membranes are non-biodegradable, they tend to persistently break down into smaller, environmentally toxic monomers when exposed to ultraviolet (UV) radiation due to sunlight [87].

The removal of the broken pieces of synthetic polymers from both the land and water is tedious and expensive [88]. Furthermore, the broken, smaller pieces can be mistaken for food by aquatic animals [87,88]. As much as recycling plastic regulations are established in South Africa, unfortunately these regulations are continually challenged. This is because of the limited plastic waste recycling infrastructure, and the plastics eventually pollute the environment [19]. In addition, the plastic waste that remains unrecycled ends up incinerated, and approximately 2.8 kg of carbon dioxide is released when 1 kg of plastic waste is burned. Other gases that are released during incineration include carbon monoxide and nitrogen oxide, and these are highly toxic to the environment [29].

Most researchers predominantly consider synthetic polymers for membrane technology suitable for water treatment applications. This is because synthetic polymers have significant chemical resistance, tensile strength, mechanical strength, and flexibility properties when compared to natural biopolymers [10,89]. However, due to their chemical resistance, these synthetic polymers degrade much more slowly and persist in the environment over an elongated period of time [89]. Consequently, some researchers have decided to blend synthetic polymers with natural polymers. Nonetheless, this does not result in completely biodegradable material.

For example, Kumar et al. [90] blended cellulose acetate with polyphenylsulfone (PPSU) membranes. Consequently, because of the resultant enhanced hydrophilicity and porosity of the membranes, their water permeability and antifouling properties were improved. In addition, Alam et al. [13] incorporated carrageenan (kCg) into PVDF membranes. The kCg concentration ranged between 0.5 wt% and 2.0 wt%. Notably, the addition of the kCg biopolymer increased the porosity and hydrophilicity of the modified membranes. Consequently, this positively influenced the modified membrane’s water permeability. Therefore, considering the variety of challenges associated with nonbiodegradable membrane materials, it becomes imperative to consider alternate biodegradable polymeric membranes.

The use of biopolymers without synthetic polymers in membrane fabrication is a promising application due to their biodegradability, optical nature, and hydrophilicity. Some other biopolymers that have been blended with synthetic polymers include chitosan [91] and cyclodextrin [92]. Furthermore, because of the improved water permeability and rejection performances of the biodegradable biopolymers, it has become vital for researchers and inventors to further explore biopolymeric applications in membrane fabrication and water treatment. Most importantly, considerations should be made towards exclusively using biopolymers for water treatment instead of blending with synthetic polymers. This can be accomplished after addressing biopolymeric application challenges such as limited mechanical strength and their solubility capacity in various solvents suitable for membrane synthesis.

## 3. Biopolymers’ Applications

Since biopolymers are predominantly hydrophilic, they are suitable for a variety of water treatment techniques such as adsorption and filtration applications. However, biopolymers tend to be more brittle and fragile, making them difficult to use under highly strenuous conditions. As such, their elasticity and tensile strength are compromised. In addition, most biopolymers are insoluble in most common solvents. Consequently, this limits their applications in various filtration applications [91,93]. Nevertheless, chemical modifications of biopolymers and blending biopolymers with synthetic polymers and nanomaterials tend to improve their flexibility and thermal stability [93]. Hence, Lu et al. [94] fabricated thermally stable silk nanofiber membranes by incorporating CeO_2_ nanoparticles. These modified membranes exhibited better tensile strength, flexibility, and elasticity properties.

Biopolymers have been considered in various forms in water treatment, as illustrated in Table 1. Biopolymers are predominantly utilized in packaging applications in South Africa [5,10,93]. Biopolymers have automotive, sports, adhesives, paints, and construction applications [5]. In addition, biopolymers have also been used in photographic films’ and filtration membranes’ synthesis and fabrication [10]. Biopolymers have also been applied in other industrial activities besides water treatment. For example, carrageenan has been used in different sectors such as drug delivery, packaging, and food additives [28,95,96], whereas cellulose has been studied in drug delivery and textiles [97]. Starch has also been explored in food packaging [98,99]; fibrinogen in drug delivery and wound healing [100,101]; silk in wound healing [102,103]; collagen in tissue engineering [104,105,106] and drug delivery [107,108,109]; pullulan in drug delivery [110], food packaging, and food stabilizing [111,112]; alginate in biomedical applications [113,114]; chitin in tissue engineering [115] and enzyme immobilization [116,117]; and collagen in tissue engineering [104,105,106] and drug delivery [107,108,109]. However, our literature search demonstrated that biopolymers have limited applications in water treatment even though they possess useful properties such as hydrophilicity, an optical nature, and an easy-to-functionalize capacity. Therefore, Table 1 reports on the applications of biopolymers in water treatment.

## 4. Biopolymeric Membranes in Water Filtration

Researchers have been developing new techniques as solutions for water treatment [6,8,30,132]. Green water treatment strategies that can enable water recycling are also being pursued [133]. Due to the incredible properties of biopolymers, it becomes ideal and attractive to utilize natural polymers towards membrane synthesis, fabrication, and production to prepare completely biodegradable membrane materials [86]. Pure biopolymers have been used on the fabrication of membranes for water treatment. These biopolymers/membranes can be utilized as adsorbents. For example, cellulose biopolymers and derivatives are being adapted for water filtration by fabricating them into ultrafiltration, nanofiltration, and osmotic membranes. Notably, these membranes are being used in the removal of contaminants such as dyes [6,8,12,40], microorganisms [8,134], heavy metals and salts [6,8,135], pharmaceuticals [8,38], pesticides, and oil/grease [8,40]. However, more work still needs to be done to improve the characteristics and performance properties of biopolymers [10].

Pandiarajan et al. [136] used orange peel-activated carbon for the removal of chlorophenoxyacetic acid herbicides from water via the adsorption–desorption technology. Consequently, this bio-sourced-activated carbon significantly adsorbed chlorophenoxyacetic acid herbicides from water. These biopolymers can be tuned into membranes. For example, cellulose polymers have been extensively utilized in water treatment membrane applications because of their plenteous polysaccharides. Hence, the polymers provide affordability and application in textile, paper [8], pharmaceutical, and membrane technology applications [8,137]. Different types of biopolymer-based membranes can be prepared such as blend membranes, nanofibrous membranes, mixed matrix membranes, imprinted membranes, and thin film composite membranes [8]. Janesh et al. [138] fabricated all biopolymer-based membranes based on chitin-glucan, chitosan-glucan, chitosan-glucan with cellulose for the removal of cationic pollutants through metal chelation. Additionally, Abdellah et al. [139] used cellulose blended with catechin membranes for the removal of DMF via solvent permeation. The membrane had a high DMF permeability of 1.2 Lm^−2^ h^−1^ bar^−1^ with a molecular weight cutoff of 500 g mol^−1^.

Primarily, the challenge with using biopolymers such as cellulose as a polymer for manufacturing membranes is that they are insoluble in common solvents. However, cellulose can be dissolved in N-methyl-morpholine-N-oxide (NMMO). In addition, cellulose can be dissolved in a dual-solvent system such as dimethylacetamide (DMAc)/lithium chloride (LiCl) [8,30,40,140], hydrazine/thiocyanate, N-methylmorpholine-N-oxide (NMMO)/water [140], and ammonium fluorides/dimethyl sulfoxide (DMSO). Fortunately, cellulose is compatible with other biopolymers, and this can help researchers create exclusive bio-based mix materials. However, pristine biopolymers have limited adsorption capacity when compared to functionalized/modified biopolymers [8]. Thus, modifying biopolymers remains ideal for enhanced adsorption–desorption capacities, increased compatibility with other biopolymers, and solubilities in various solvents.

### 4.1. Progress in the Preparation and Functionalisation of Biopolymers for Water Treatment

In general, the disadvantage and limitation of biopolymers in water treatment applications is their mechanical instability [19,141]. The poor mechanical properties of biopolymers restrict their application in water filtration because of the high pressures that is used during water filtration. Hence, the biopolymeric membranes can break [141]. However, these properties can be enhanced through modification processes [10]. Consequently, the modifications cannot only improve the mechanical stability, but also the antifouling properties, self-cleaning capacities, and water permeability of the resultant membranes [1]. Thus, more work has been done to understand the effect of blending various types of synthesized or natural polymers with biopolymers to obtain maximum separation performance, such as higher flux, swelling capacity, permeation, and better selectivity [8].

**Scheme 1:**
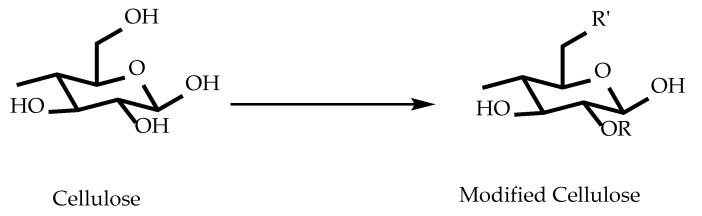
General scheme of cellulose modification where R and R’ are functional moieties [142,143,144,145,146].

Since biopolymers lack sufficient adsorptive–desorptive affinity towards organic pollutants such as cationic dyes, it is required that new functional groups are introduced, as illustrated in Scheme 1 using cellulose as an example. This includes the introduction of functional groups such as sulphur, amine, and hydroxyl, and carboxylic groups because of their higher affinity towards micropollutants. Notably, the ligands containing these functional groups can enhance the selectivity of modified membranes and significantly participate in the adsorption–desorption processes of contaminants [12]. The hydroxyl group on the cellulose backbone also coordinates with charged pollutants such as salts and heavy metals via electrostatic attraction, ion exchange, van der Waals forces, and hydrogen bonding [135].

The introduction of the new functionalities to the backbone of the biopolymers such as cellulose has been achieved through various reactions. For example, cellulose modification is accomplished through the modification of the hydroxyl group along the cellulose chain [6,10,144,147]. Such modifications can be accomplished through cationization, phosphorylation [6,8], (2,2,6,6-tetramethyl-piperidin-1-yl)oxyl (TEMPO) oxidation [6,8,148], etherification, grafting, halogenation, carboxymethylation, sulfoethylation, sulfonation, aminoguanidine, ozonation, thiolation [6], esterification [6,10], acetylation [10], and amination [6,143,144,149]. The mechanisms of these reactions replace the proton of the hydroxy group of cellulose with functional groups, resulting in enhanced physico-chemical properties of the membrane [150].

Maleš et al. [12] modified cellulose with different functionalities into carboxymethyl cellulose (CMC), cellulose nanofibrils (CNF), and bacterial cellulose (BC) membranes. Consequently, these modified membranes efficiently removed azo and anthraquinone dyes from wastewater. The CMC and CNF membranes exhibited a 100% removal efficiency against anthraquinone dye. However, all these modified biopolymeric membranes had low removal efficiencies against azo dyes. Biopolymers are useful in the removal of these pollutants because of their significant adsorption capacity. Most importantly, the biopolymers can be modified/blended into various derivatives to possess desired properties useful for water treatment, as discussed in Table 2.

### 4.2. Removal of Organic Pollutants with Hybrid Biopolymeric Membranes

**Figure 4:**
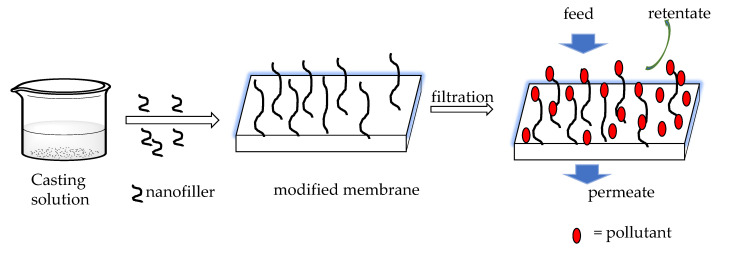
Illustration of polymer modification and filtration.

Besides the modification of the biopolymers, nanomaterials can also be embedded into the biopolymer matrices to increase the performance and characteristic efficiencies of the biopolymeric membranes [19,141]. As such, several modification methods have been explored to functionalize biopolymeric membranes in pursuit of introducing functionalities and characteristics. For example, biopolymers were surface grafted, coated, and doped with nanoparticles [1]. In addition, the incorporation of nanofillers has also been adopted for biopolymeric membranes. The sizes and types of the nanofillers can influence the properties of the biopolymeric membranes. Notably, varying the nanofiller surface area-to-volume ratio improves the properties of the membrane such as their catalytic activity, adhesion properties, electrical resistivity, and chemical reactivity [24,141] and this has an impact on the effectiveness of the membrane, as illustrated on Figure 4.

The nanoparticles increase the hydrophilicity of the biopolymeric membranes as well as their pollutants’ removal efficiencies. As a result, this significantly increases the antifouling capabilities of the modified biopolymeric membranes [19,23]. Notably, nanomaterials have a large surface area. Consequently, this presents abundant active sites that remain exposed for interactions with pollutants. Furthermore, the reactivity and selectivity of nanostructured biopolymers towards the pollutants get increased as well as modified membrane’s effectiveness [23]. The different sizes and shapes of nanofillers can determine the interaction capacity of the modified membranes with pollutants [24]. However, the size of the nanoparticles can be affected by various factors such as concentrations of reagents, mole ratio of reactants, reaction methods, and reaction time [156].

Nanofillers can be grouped into inorganic material, organic material [25,141], carbon nanostructures [141], and hybrid material [25]. For instance, the carbon nanostructures include carbon nanotubes and graphene sheets and inorganic nanofillers such as metal oxides and metals, among others [141]. Furthermore, organic nanofillers such as biopolymers such as cellulose, chitosan [141], and carrageenan have been used to modify synthetic polymeric membranes. Notably, metal oxides such as TiO_2_, tungsten oxide (WO_3_) [1,21], SnO_2_ [1], Ag_2_O [156], ZnO [21,156] and silver phosphate (Ag_2_PO_4_) [21] have been used to modify biopolymeric and synthetic membranes.

These nanoparticles are synthesized via various techniques. For example, Siddiqui et al. [156] synthesized silver oxide nanoparticles via the capping method while silver chloride nanoparticles were synthesized by simple precipitation of silver nitrate solution. Researchers synthesized Ag_2_O nanoparticles via the chemical precipitation reactions [157,158,159]. Other nanoparticles have been synthesized via the precipitation technique such as ZnO nanoparticles [160,161,162]. There are several nanofillers that have been explored in cellulose-based membrane production such as carbon nanotubes, nanoparticles, and nanosheets [8] as well as carbon-based nanomaterials such as the graphitic carbon nitrides.

Nanofillers can introduce new functionalities when incorporated into the biopolymeric membranes, improving their thermal and mechanical properties [19,25,141], hydrophilicity, porosity [19,25], antimicrobial and antioxidant properties [8,24,25,141], photocatalytic properties [8,24,25], adsorption–desorption properties [8,24], and barrier properties [141]. The nanofiller impacts on biopolymeric membranes and synthetic performances are reported in Table 3.

A study done by Xie et al. [167] demonstrated the effect of the nanofillers in starch-based membranes. Graphene oxide/Bi_2_WO_6_ (GBW) was added onto starch membranes as a photocatalyst for ethylene degradation. The amount of GO on the starch membranes determined the effectiveness of the photocatalysis. When the addition of GO was 0.5%, GBW/starch composite film showed the strongest visible light degradation activity for ethylene, and the rate constant K’ was 9.91 × 10^−4^ min^−1^, 4.4 times that of pure Bi_2_WO_6_, whereas the 0.25% GO in GWB was less effective. The tensile strength of the membrane was greatly improved by the addition of GBW. As the amount of GO was increased, the tensile strength was also increased. A tensile strength of 23.19 MPa was achieved when 1% of GO of GBW composite was used, as a result of the toughening effect on the polymer film and the hydrogen bonding between the polar groups on the surface of GBW and the hydroxyl groups in the starch molecules having a major role in improving the mechanical properties of the composite membrane [167].

Furthermore, Zhao et al. [132] prepared cellulose acetate/chitosan composite membranes that were enriched with activated carbon. Consequently, these modified membranes exhibited an enhanced water permeation flux of 9.09 × 10^3^ L m^−2^ h^−1^, coupled with 99.6% rejection capacity of bisphenol A pollutants. This was accomplished at low pressures of 0.1 MPa. Such performances were attributed to the improved hydrophilicity and adsorption capability that was introduced by the activated carbon. Han et al. [165] incorporated multi-walled carbon nanotubes (MWCNTs) with various diameters (10–20 nm, 20–40 nm, and 40–60 nm) into cellulose membranes. Their incorporation enhanced the antifouling and separation performances of the membranes. Notably, as the outer diameter of MWCNTs increased, the antifouling properties of the modified membranes also improved.

Abdelhameed et al. [168] reported the preparation of copper-benzene-1,3,5-tricarboxylate (BTC) metal-organic framework (Cu-BTC) on cotton (Cu–BTC@cotton) composite, which was intended for the removal of organophosphorus insecticides (ethion) from water. In their study, as the amount of copper on the composite membranes increased, the adsorption capacity also increased. For example, the pure cotton adsorption capacity was 10 mg/g, 26.7 mg/g for the 5% Cu-BTC@Cotton, and 182 mg/g for 10% Cu-BTC@Cotton. The removal percent of ethion exceeded 97% for the 10% composite after 120 min. In a continuation to their study, Abdelhameed et al. [169] used CuBTC on cellulose acetate (Cu-BTC @CA) for the removal of another pesticide (dimethoate). The grafting of Cu-BTC onto CA membranes enhanced the adsorption from 207.8 mg/g to 282.3–321.9 mg/g. Of note is that after five cycles of adsorption–desorption, the adsorption capacity of the composite membrane was decreased by 22.5%. In another study, Garba et al. [170] were able to adsorb prometryn (Pr) herbicide using microcrystalline cellulose incorporated with copper, and obtained an adsorption capacity of 97.80 mg/g at ambient temperature, whereas at 50 °C the adsorption capacity increased to 119.70 mg/g.

**Figure 5:**
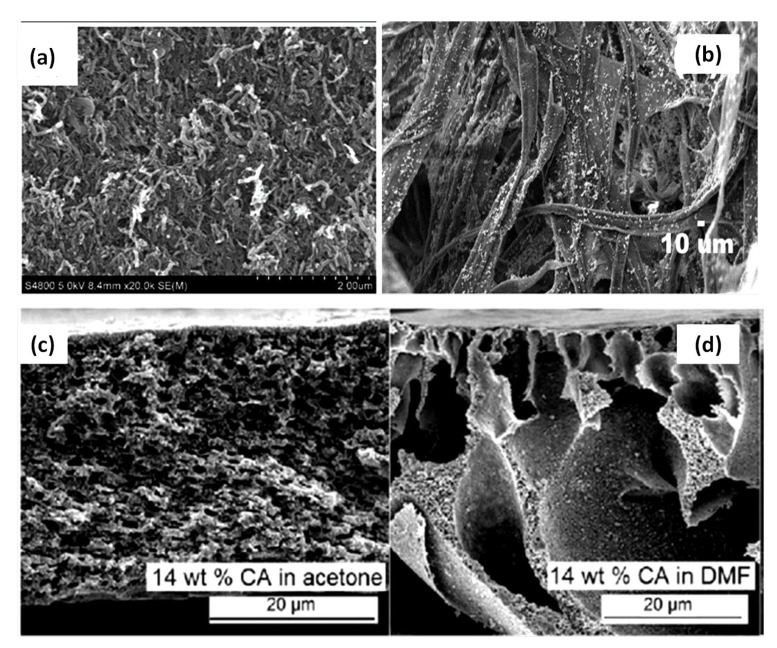
SEM micrographs of; (**a**) cellulose membranes incorporated with MWCNTs; (**b**) cellulose foam filter paper impregnated with ZnO nanoparticles; and (**c**) and (**d**) cross-sections of cellulose acetate membranes prepared from solutions in acetone and DMF, respectively. The micrographs were retrieved from literature: (**a**) [165], (**b**) [157], and (**c**) and (**d**) [171].

The solvent used for fabrication and the nanofiller type and fabrication techniques determines the resultant membrane morphology, as illustrated in Figure 5. Cellulose acetate membranes had larger pores when DMF was used as a casting solvent compared to when acetone was used. This can be attributed to the difference in evaporation rates [171].

On the other hand, Ao et al. [30] immobilized La(OH)_3_ nanosheets onto cellulose- based membranes and this effectively improved the membranes’ dye removal capabilities. For instance, the modified membrane’s adsorption capacities were found to be 624 mg/g on Congo red (CR) compared to only 260 mg/g Congo red rejection of the unmodified cellulose-based membrane. This enhanced performance was attributable to ion exchange capacities on the modified membranes introduced by the La(OH)_3_ nanosheets. This was explained by the positively charged Congo red dye. The positive charge enabled the dye’s easier attachment to the O- anion of the OH functional groups of both the cellulose and La(OH)_3_ nanosheets.

Cheng et al. [34] also tested the cellulose nanofibrous membranes incorporated with polydopamine on the adsorption of MB. These membranes exhibited an adsorption capacity of 88.15 mg/g at room temperature under pH of 6.5, on an adsorption cycle of 30 h. The increased adsorption capacity was attributed to the presence of amines brought by the polydopamine on which the cationic dyes tend to adsorb. These results reflect that cellulose-based membranes can be used for water filtration with optimum membrane performances for the treatment of organic pollutants in contaminated water depending on the polymer/membrane modifications. On another note, in an attempt to improve the hydrophilicity and rejection capacity of cellulose acetate, De Guzman et al. [172] incorporated polydopamine-sulfobetain nanoparticles, which improved the hydrophilicity of the membrane. These modified membranes had higher water flux (up to 583.64 ± 25.12 L m^−2^ h^−1^) and a high rejection of oils (hexane, toluene, food oil, diesel, dodecane) [172].

Another way of modifying membranes to improve the physico-chemical properties is through crosslinking. For example, Zhao et al. [37] crosslinked P84 copolyimide membranes with polyethylenimine for the removal of two antibiotics (cefadroxil and enrofloxacin) using polyethylenimine cross-linked nanofiltration. Different molecular weight PEIs of 800, 2000, 25,000, and 750,000 g·mol^−1^ were utilized. Among these four membranes, the PEI-25k membrane had a high permeate flux compared to its counterparts. The crosslinking affected the membrane morphology. The P84 copolyimide membrane with no polyethylenimine had a thin and loose skin layer on top of a porous sublayer, whereas the P84 copolyimide modified with 25 k polyethylenimine had a thicker and denser skin layer and was less porous. This is because during the crosslinking the PEI molecules diffused into the membrane, hence reducing the porosity of the membrane. The crosslinked membranes had 90% rejection of enrofloxacin molecules at pH 3–4, which was due to the electrostatic repulsion between the positively charged enrofloxacin and membrane, whereas the enrofloxacin retention was low due to the electrostatic attraction between the positively charged membrane surface and negatively charged carboxyl groups of enrofloxacin [37].

Dodero et al. [173] investigated the use of phosphate ions (i.e., Na_2_HPO_4_) and ethylene glycol diglycidyl ether as crosslinkers on chitosan nanofibrous membrane. The success of the crosslinking was measured against morphological, mechanical, water-related, and biological properties. The use of phosphate ions crosslinked membranes were smooth, homogenous nanofibers with an average size of 190 nm, whereas the ethylene glycol diglycidyl ether crosslinked membranes were rougher with average size of 270 nm, which was bigger than their counterparts. The mechanical study revealed that the phosphate ions crosslinked membranes showed enhanced mechanical performances, as well as greater water vapor permeability and hydrophilicity, with respect to the chemically crosslinked ones. The tensile strength and Young modulus of the phosphate ions crosslinked membranes were almost twice those of the ethylene glycol diglycidyl ether crosslinked membranes [173].

Of note, the membrane specifications such as pore size and membrane thickness as well as the properties of nanoparticles incorporated are important in determining the effectiveness of the membrane. For example, Liu et al. [174] prepared sulfated cellulose nanocrystal membranes with an average pore size of 0.22 µm and were able to reject tetracycline hydrochloride with a molecular weight of 480.90 g/mol. Additionally, Li et al. [175] rejected tetracycline with a molecular weight of 444.435 g/mol and methyl orange via photocatalysis with a cellulose acetate membrane containing H_4_SiW_12_O_40_ and a pore size of 301.1 nm. Tetracycline was also degraded by Shi et al. [176] using g-C_3_N_4_/BioBr photocatalysts on carbon fibers.

### 4.3. Challenges on the Implementation and Application of Biopolymers in Water Treatment

The major hindrances for the development, upscaling, and reaching the market of biopolymeric membranes are their solubility and mechanical strength challenges. Biopolymers are insoluble in common solvents, which will make the production of the membranes pricey [91,93]. Additionally, biopolymeric membranes are mechanically weak compared to their nonbiodegradable counterparts, hence the need of blending, crosslinking, and introduction of nanofillers [177]. Even then, there is limited reporting on the upcycling and recycling of biopolymer membranes after use to determine the resilience in water treatment. Therefore, researchers still must address the solubility and mechanical strength challenges of biopolymers for maximal applications in adsorptive and membrane technology applications.

Khaless et al. [178] demonstrated the possibility of recycling membranes in an attempt to apply them in the clarification of wet-process phosphoric acid. They demonstrated the possibility of recycling membranes in an attempt to use them in the clarification of wet-process phosphoric acid. In this study, they proved the potential of recycling spent reverse osmosis (RO) membranes. The RO membranes were transformed into microfiltration (MF) membrane by stripping them with NaOH, KMnO_4_, and KMnO_4_/NaOH. The MF treated with NAOH were able to reject 61% of organic matter and 70% of suspended particles, which was better than the KMnO_4_-regenerated membranes, which had a 61% rejection of organic matter and 54% rejection of suspended particles. When it comes to membrane permeance and flux, it was reported that all the regenerated membranes showed an improvement from that of spent RO membranes. The previously spent membrane had a permeability of less than 1 L m^−2^ h^−1^ bar^−1,^ whereas the regenerated membranes using NaOH, KMnO_4_, and KMnO_4_/NaOH had a permeability of 40 L m^−2^ h^−1^, 45 L m^−2^ h^−1^, and 43 L m^−2^ h^−1^ bar^−1,^ respectively. The membrane permeability remained relatively the same after 7 days. Lastly, the wet-process phosphoric acid flux of the regenerated membranes was 43 L m^−2^ h^−1^, 54 L m^−2^ h^−1^, and 53 L m^−2^ h^−1^ for NaOH-, KMnO_4-_, and KMnO_4_/NaOH-treated membranes. Likewise, the flux did not change significantly since it changed into 44 L m^−2^ h^−1^, 56 L m^−2^ h^−1^, and 51 L m^−2^ h^−1^ after 7 days of use on the respective membranes.

In addition, Dai et al. [179] recycled used biopolymers for fabrication of highly permeating, thin-film composite membranes. Dai et al. used already-used biopolymers to fabricate high-permeance, thin-film composite polyamide membranes. In this study, fouled microfiltration membranes were upcycled for fabricating polyamide (PA) thin-film composite membranes via interfacial polymerization (IP) purposes. The thin film composite membranes had an average water permeability of 30 L^−1^ m^-2^ h^−1^ bar^−1^ and 95 % rejection of Na_2_SO_4_. Furthermore, some researchers [180,181] have demonstrated that biopolymers are biodegradable. For example, Fenyvesi et al. [181] demonstrated the degradation of cyclodextrin in soil and recorded more than 90% degradation after 178 days. However, with all these promising decomposition performances, there is still limited appetite and efforts towards adopting biopolymers for various water treatment applications. Therefore, after use, the biopolymeric membranes can be used as compost under specific conditions such as temperature and moisture. Thus, more efforts should be towards comprehensively studying the decomposition rate of biopolymers to add value after use in compost creation.

## 5. Conclusions

Organic micropollutants are a threat to the environment across terrestrial and aquatic environments. Therefore, developing efficient, sustainable, and techno-economically membrane-based filtration systems can prevent short- and long-term toxic effects of the organic micropollutants. However, since synthetic polymeric membranes, which are currently being used to develop water treatment membranes, are non-biodegradable, considerations should be made towards biodegradable polymeric materials, to avoid secondary pollution. This means that biopolymers such as cellulose should be considered, especially because they are entirely biodegradable, abundant in nature, cost-effective, and affordable. Most importantly, natural biopolymers are unique because they are renewable, sustainable, nontoxic, and biocompatible. The blending technique has observed minimal applications because biopolymers are insoluble in common organic solvents and mechanically weak compared to nonbiodegradable membranes. As such, more research work should be carried out to address this limitation. In addition, the incorporated nanoparticles and functionalization of the biopolymers should be considered. Concerning biopolymeric membranes, to date, membrane antifouling properties, resistance to pH, and stability under a variation of operating pressures and temperatures without any interference with the water flux and rejection capabilities have observed limited optimization. Thus, further investigations with this regard are required, to achieve practically sustainable direct biopolymeric membrane filtration operations. Thus, depending on the modification protocols of biopolymers such as increasing the aspect ratio, improving membrane’s mechanical and enhancing their physico-chemical properties should be considered [182].

## Figures and Tables

**Table 1:** Reported applications of biopolymers in water treatment.

Polymer	Nature of Material	Application	References
Carrageenan	Aerogel	Water treatment—adsorption–desorption of heavy metals	[118]
Cellulose	Aerogel	Water treatment—adsorption–desorption of heavy metals, dyes, and oils	[119]
	Film/membrane	Water treatment—various pollutants	[90,120,121]
	Adsorbents	Adsorbent of Zn(II), Co(II), Cd(II), and Ni(II)	[122]
Chitosan	Nanofibrous membrane	Water treatment—removal of heavy metals	[123]
Cyclodextrins	Membranes	Water treatment: desalination	[92]
Starch	Hydrogel	Nanoadsorbents for the removal of cationic dyes from water	[124]
Silk	Aerogels Fibrous membrane	Water treatment: removal of oil	[125,126,127]
	Membranes	Water treatment: dye degradation	[128]
Pullulan	Films, gels	Waste treatment: biosorption of heavy metals	[111,112]
Alginate	Beads	Water treatment: adsorption of heavy metals	[129]
Chitin	Nanofibrous membranes	Water treatment: removal of organic hydrophobic organic contaminant.	[130,131]

**Table 2:** Several effective uses of biopolymeric membranes in water treatment.

Membrane Types	Membrane Materials	Pollutant Treated with Membranes	Membrane Performance	References
Thin Film Composite (TFC) by interfacial polymerization	Catechin/cellulose	Amido black dye (617 g/mol)	92% rejection of dye was reported	[139]
TFC membrane	Cellulose/polydopamine	MgSO_2_	Stable membranes with a rejection capacity of up to 75.6% and water flux of 25.06 L m^−2^ h^−1^ at a pressure of 0.4 MPa was reported.	[27]
NF membranes	Cellulose acetate blended with Nicotiana tabacum ash and Fe0 nanoparticles. (ACA@Fe0)	Congo Red (CR), Methyl Blue (MB), Methyl Orange (MO), 4-Nitrophenyl phosphate (4NP)	CR, MB, MO dyes were reduced using the ACA compared to ACA@Fe0; however, the 4NP was reduced using the ACA@Fe0	[14]
NF membranes	ZIF-8/chitosan/Polyvinyl alcohol	RG dye	142.85 mg g^−1^ of dye was adsorbed	[151]
NF membranes	Bacterial cellulose with MOFs	Nitrobenzene	Water permeation of 10.85 L m^−^^2^ h^−1^ psi^-1^ rejection of nitrobenzene (68.6%)	[152]
NF membranes	Chitosan with oxidized starch and silica	Blue 71 and Red 31	Good thermal stability and swelling properties. Adsorption capacity increased as the pH increased.	[153]
NF nanofibrous membranes	Hyper-crosslinked cyclodextrin membranes	MB, Safranin O, rhodamine B, MO, methyl red, CR, rose bengal, and direct red 80	adsorption capacity of above 180 mg g^−1^	[154]
NF nanofibrous membrane	Cross-linked β-cyclodextrin	steroid hormone: estradiol	removal efficiency of 75% after only 180 min and reaching the saturation after 5 h with 80% removal	[155]
TFC via coating	Filter paper coated with activated cellulose (cotton)	MB	98% rejection at lower concentrations (5–10 ppm), 89% rejection at 20 ppm 78% rejection at 100 ppm	[40]
UF nanofibrous membranes	La(OH)_3_@cellulose	Oils: Hexane, cyclohexane, toluene, pump oil, crude oil, petroleum ether; Dyes: CR, MB, MO	High water flux (5897.7 L m^−2^ h^−1^), which is 2 times greater than cellulose membranes. Above 90% rejection of oils was observed. 91.2% of CR was adsorbed effectively, whereas MB and MO were not adsorbed. Membrane was selective to CR. CR was adsorbed from a CR/MO mixture	[30]
UF nanofibrous membrane	Deacylated cellulose/acetate with polydopamine (DA@PDA)	MB	88.15 mg/g was adsorbed, which is about 9 times higher than the adsorption observed on DA.	[34]
UF nanofibrous membrane	Cellulose nanofibril (CNF), Carboxymethylated cellulose (CMC), Bacterial cellulose (BC)	Anthraquinone dye Azo dye	100% rejection of anthraquinone dye with CNF and CMC membranes and 24.3% rejection with BC. Less than 10% rejection was observed on azo dye on all membranes	[12]

**Table 3:** The effects of nanofillers on biopolymeric and synthetic membranes’ behavior.

Nanofillers	Material	Film Property	Effect on Membranes	References
Sorbitol	Starch films	Thermal properties, morphology	The sorbitol decreased the onset temperature for the films, hence improving the sealing process. Uniform thickness at high concentration of sorbitol.	[163]
Sorbitol/glycerol	Alginate films	Mechanical properties	The plasticizers increased the plasticizing effect, hence increasing the mechanical strength.	[164]
Multiwalled carbon nanotube (MWCN)	Cellulose Membranes	Antifouling properties	Improved antifouling characteristic as the outer diameter of MWCNT increases.	[165]
Chitosan	Bacterial cellulose/poly(vinyl alcohol) membranes	Mechanical properties	Improved mechanical properties; reported tensile strength was 130.55 ± 9.42 MPa.	[166]
	Polysulfone membranes	Antifouling properties	Improved antifouling properties with smoother surface after coating with coating of CS-Ag nanomaterials	[16]
Bisphenol A-type-benzoxazine (BATB)	Amino cellulose (AC)	Mechanical and chemical properties	Increased tensile strength BATB was more thermally stable than AC	[144]
ZnO, CuO, Ag_2_O nanoparticles	Cellulose membranes	Antimicrobial properties	Antimicrobial composite membranes were achieved against *E. Coli*, *P. aeruginosa*, *B. subtilis*, and *B. cereus strains*. Ag_2_O/cellulose had the highest efficacy.	[157]
Carrageenan	PVDF membranes	Hydrophilicity	Increased permeability and water flux due to increased porosity. 71% MO rejection compared to 25% on pure PVDF	[13]
TiO_2_ nanoparticles	PES membranes	Hydrophilicity, thermal, and mechanical properties	Increased hydrophilicity as TiO_2_ nanoparticle concentration increased. Maximum flux obtained at 4–5 wt% of TiO_2_ nanoparticles. Thermally stable composites with higher breaking strength and low elongation ratio to PES were produced	[83]

## Data Availability

Data sharing not applicable.
